# Tomato juice intake suppressed serum concentration of 8-oxodG after extensive physical activity

**DOI:** 10.1186/1475-2891-11-29

**Published:** 2012-05-02

**Authors:** Mats Harms-Ringdahl, Dag Jenssen, Siamak Haghdoost

**Affiliations:** 1 Centre for Radiation Protection Research (CRPR), Department of Genetics, Microbiology and Toxicology, Stockholm University, SE-106 91, Stockholm, Sweden

**Keywords:** Reactive oxygen species, ROS, Free radicals, Exercise, Lycopene, Tomato juice, Life style, ELISA, hMTH1, 8-oxo-dG

## Abstract

**Background:**

DNA is constantly exposed to reactive oxygen species (ROS), spontaneously arising during the normal oxygen metabolism. ROS may result in temporary as well as permanent modifications in various cellular components such as lipids, proteins and DNA, which may have deleterious consequences. Demonstrating that a dietary supplementation of antioxidants can reduce oxidative DNA damage may provide evidence for the value of such supplementation in prevention of cancer and age related diseases.

**Findings:**

The present study was conducted to address whether tomato juice protects against ROS induced by extensive physical exercise in untrained individuals. As a marker of oxidative stress, serum levels of 8-oxodG were monitored using a modified ELISA. An intervention was performed involving 15 untrained healthy subjects who performed a 20 min physical exercise at 80% of maximum pulse using an ergometer bicycle. Blood samples were taken before and one hour after the exercise. The procedure was repeated after 5 weeks with a daily intake of 150 ml tomato juice and followed by a 5 weeks wash-out period and another 5 weeks with a daily intake of tomato juice. The results indicated that a daily intake of tomato juice, equal to 15 mg lycopene per day, for 5 weeks significantly reduced the serum levels of 8-oxodG after an extensive physical exercise.

**Conclusion:**

These data strongly suggest that tomato juice has a potential antioxidant effect and may reduce the elevated level of ROS induced by oxidative stress.

## Findings

It has been suggested that dietary antioxidants reduce the level of oxidative DNA damage induced by reactive oxygen species. However, there are limited in vivo studies which support this hypothesis as a number of epidemiological studies showed no such effect following dietary supplementation with carotenoids, vitamin C, or E [1,2]. Urinary concentration of 8-hydroxy-2′-deoxyguanosine (8-oxodG, a base damage formed by reactive oxygen species) has been used as a non-invasive biomarker of oxidative DNA base damage in a number of studies [3,4].

Reactive oxygen species (ROS) have been suggested to play an important role in mutagenesis, carcinogenesis, and aging processes. ROS can react with different cellular components e.g., proteins, lipids and nucleic acids, and give rise to chemical modifications. Under normal conditions cellular antioxidant enzymes and other antioxidants in the cell detoxify elevated levels of ROS and minimize damage to intracellular components. However, under extensive physiological activity ATP consumption will increase followed by increased oxygen consumption and, as a consequence, the production of ROS will increase [5]. Healthy and/or well-trained persons and vegetarians seem to have increased protection against ROS-induced damages [6]. It suggests that regular physical exercise [7] and a diet rich in antioxidant [8] may have a protective effect towards ROS-induced damage, in particular DNA base damage. One of the frequently studied DNA base damages is 8-oxodG. Highly effective repair mechanisms are operating both on DNA (e.g., hOGG1) and the nucleotide pool (hMTH1) to remove 8-oxodG/8-oxodGTP from the cell [9,10]. Previously, we have shown that extracellular 8-oxodG originates from the nucleotide pool and may serve as a sensitive marker of oxidative stress [11]. We have developed an ELISA method that allows the determination of the very low concentrations of 8-oxodG present in human blood serum [11-13].

In the present study we aimed to investigate the protective effect of tomato juice intake towards ROS induced by 20 min of extensive physical exercise. A novel finding in the present intervention study was that the level of 8-oxodG in human blood serum was increased significantly after 20 min acute physical activity possibly caused by an increase of the intracellular ROS level. No increase was observed when individuals had been drinking 150 ml tomato juice per day during a period of 5 weeks suggesting that the intracellular nucleic acids and, in particular, the nucleotide pool were unaffected and well protected from the deleterious effect of ROS. The intervention study support the hypothesis that antioxidants (e.g. lycopene) supplied from tomato juice may protect against oxidative stress induced by extensive physical exercise.

## Materials and methods

In this study 15 healthy and untrained donors (Table 1) were asked to drink 150 ml tomato juice per day in 2 periods of 5 weeks each as follows: 5 weeks with tomato juice, 5 weeks without tomato juice and 5 weeks with tomato juice. Blood samples were collected before and after start of each period during the intervention study. At the day for blood collection, the individuals were asked to do 20 min physical exercise with 80% max pulse using an ergometer bicycle. To calculate the individual maximum pulse, the following generally accepted formula was used: 220 - age =maximum pulse. Blood samples were collected before and after exercise. The serum level of 8-oxodG was analyzed as a marker of oxidative stress. Of note, this intervention study was designed according to recommendations which have been published by Loft and co-workers [14]. The study was performed in accordance with the ethical standards and approved by the Swedish Ethical Committee at the Karolinska University Hospital (Dnr 03–621).

**Table 1:** Main characteristics of the donors

**Donors**	**Age**	**Sex**	**Smoking**	**Vegetarian**	**Vitamins**	**Background diseases**
1	30	F	No	no	no	no
2	28	M	No	no	no	no
3	35	M	No	no	yes	no
4	28	F	No	no	no	no
5	29	F	No	no	yes	no
6	46	M	No	no	no	no
7	29	M	No	no	no	no
8	24	F	No	no	yes	no
9	27	M	No	no	yes	no
10	25	F	No	no	no	no
11	25	F	3-5 cig/day	no	no	no
12	27	M	No	no	no	no
13	43	F	No	no	no	no
14	38	M	No	no	no	no
15	24	M	No	no	no	no

## Analysis of 8-oxodG in blood serum

Just before and one hour after each training session (20 min cycling), blood samples were collected in tubes without anticoagulant. After complete coagulation of the blood samples, the blood serum was isolated. 8-OxodG concentration of blood serum was measured using ELISA as described previously [11,15]. The ELISA kit was provided by Health Biomarkers Sweden AB. Briefly, 1 ml blood serum was purified using a C18 solid phase extraction column (Varian, CA) according to a previously published method [16]. This step is necessary to remove products other than 8-oxodG which could cross-react with the monoclonal antibody used in the kit. A standard curve for 8-oxodG (0.05 - 10 ng/ml) was established for each plate covering the range of 8-oxodG in the samples. One sample in each experiment was mixed with 1 ng 8-oxodG before purification which served as internal standard. Validation of the modified ELISA method was performed by HPLC-EC (r^2^: 0.87, p < 0.05) [15]. Comparisons between the ELISA and the HPLC-EC methods showed a linear correlation at the concentration range found in the human blood serum [15]. There was no correlation between ELISA and HPLC-EC when unfiltered samples were used.

## Determination of the lycopene content in tomato juice

The lycopene content in tomato juice was determined spectrophotometrically essentially as described in reference by Fish et al. [17]. The concentration of lycopene was calculated using the molar extinction coefficient of lycopene in hexane (17.2 × 10^4^ M^-1^ cm^-1^).

## Statistical method

Student’s t-test was used for testing statistical significance for serum levels of 8-oxodG. A p-value below 0.05 was deemed as significant.

## Results

The participants were asked to have a daily intake of 150 ml tomato juice (containing 0.1 mg lycopene per ml juice) for 35 days in 2 interventions with a wash-out period between these periods. The main characteristics of the donors are presented in Table 1. Blood samples were collected day 0 (period A), day 35 (end of period B), day 70 (end of period C; washout) and day 105 (end of period D). Two blood samples were taken at each occasion, one before and the second 60 minutes after the 20 min physical exercise. The serum level of each participant was compared to his/her own control value. Thus, each participant served as his/her own control.

**Figure 1:**
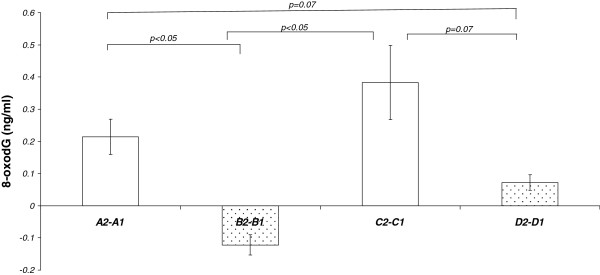
**Mean values of the individual changes in 8-oxodG serum concentrations (values before exercise are subtracted from values after exercise) after 20 minutes of exercise.** White bars show the changes when exercise was performed without previous tomato juice supplementation. Dotted bars show the values after five weeks of lycopene supplementation. For bars A2-A1 and B2-B1 n = 15 and for C2-C1 n = 11 while for D2-D1 n = 9.

**Figure 2:**
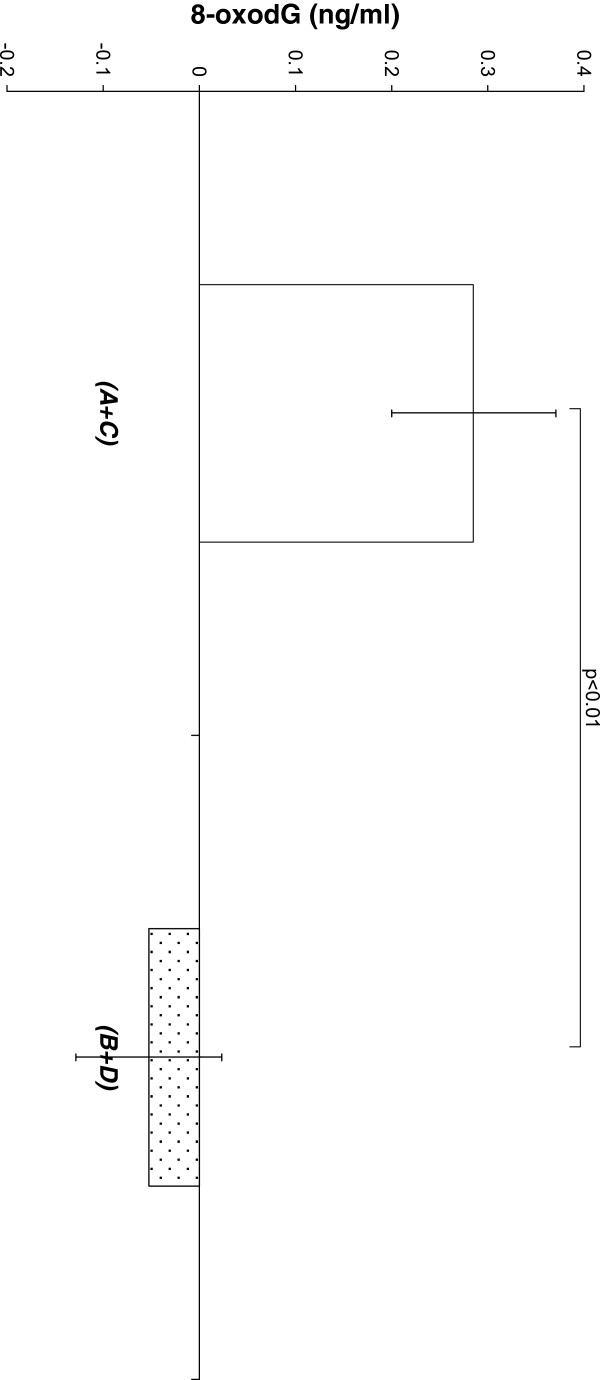
**Shows mean values of individual changes of 8-oxodG level after periods without(mean of A and C periods; n = 26) and after periods with tomato juice intake (mean of B and D values; n = 24) before and after exercise respectively.** Bars show means ± SE.

Table 2 shows the concentrations of 8-oxodG in serum at the different sampling times during the intervention study. The background levels (before exercise) of 8-oxodG did not deviate significantly during the interventions. As shown in Table 2, 20 min extensive physical activity increased the level of 8-oxodG in serum above the background level by an average of 42% (from 0.52 up to 0.74 ng/ml). In contrast, following 5 weeks (B) intake of 150 ml tomato juice (corresponding to a daily intake of 15 mg lycopene) the levels of 8-oxodG remained essentially unchanged after compared to before the exercise. After the 5-week washout period (C), the level of 8-oxodG increased again (84%) after exercise in average from 0.45 up to 0.83 ng/ml. After the second period of tomato juice intake (D), the levels of 8-oxodG after exercise were almost the same as before the exercise (from 0.31 up to 0.39 ng/ml).The results are summarized and presented in Table 2 and in Figures 1 and 2.

**Table 2:** Concentration of 8-oxodG before and after 20 min physical activity (mean ± SE) in blood serum after periods with and without tomato juice intake

	***without TJ***		***5 weeks with TJ***		***5 weeks without TJ***		***5 weeks with TJ***	
8-oxodG ng/ml	A1	A2	B1	B2	C1	C2	D1	D2
	0.52±0.11	0.74±.0.14	0.56±0.13	0.46±0.06	0.45±0.08	0.83±0.16	0.31±0.06	0.39±0.06

A1, A2, B1 and B2; n=15. For C1 and C2; n=11 while for D1 and D2; n=9.

*TJ: tomato juice*.

*Samples A1, B1, C1 and D1: the level of 8-oxodG before 20 min physical activity*.

*Samples: A2, B2, C2 and D2: the level of 8-oxodG 1h after 20 min physical activity*.

The data in Figure 2 are the average changes of 8-oxodG induced by physical activity (the concentration of 8-oxodG before exercise is subtracted from the concentration after activity exercise) without tomato juice intake (periods A and C) and with tomato juice intake (periods B and D).

As shown in Table 1, four subjects took vitamin supplementation. Also when these subjects were excluded from the statistical analysis, the effects of tomato juice intake remained significant.

## Conclusion

Based on the results from this intervention study it is concluded that 8-oxodG is significantly increased in blood serum of 15 healthy donors after an acute physical exercise, suggesting that there is a positive correlation between 8-oxodG in serum and the intracellular ROS production. The study also demonstrates that 150 ml tomato juice intake (15 mg lycopene) per day significantly protects the nucleotide pool from ROS produced in response to extensive physical activity. The proposed explanation for the observed results is that ROS induced by extensive physical activity react with intracellular dNTP and give rise to production of 8-oxodGTP. 8-OxodGTP is excreted from the intra- to the extra-cellular matrix by the action of hMTH1 protein [18] to inhibit its incorporation into the DNA. Lower levels of 8-oxodG in serum during tomato juice intake show that tomato juice intake protects dNTP from ROS induced modification.

It is important to mention that beside lycopene tomatoes also contain vitamin C, tocopherols and polyphenols [19]. It has been shown that among all antioxidants (in particular carotenoids) present in tomato juice, lycopene is the most abundant and stable during industrial food processing [19]. Vitamin C and tocopherols in fresh tomato are destroyed by heating during food processing. Not much is known about the polyphenols in tomato juice [19]. Therefore, we believe that the antioxidant activity of tomato juice is primarily due to its content of lycopene.

It might be hypothesized that long term intake of tomato juice may reduce oxidative stress levels in patients with enhanced level of oxidative stress, for example, patients with diabetes, cardiovascular diseases or inflammation. A study is in progress to test the hypothesis on patients with diabetes using the experimental model system described with 8-oxodG as biomarker.

## Competing interests

Authors have no competing interests.

## Authors’ contributions

SH served as Principal Investigator and contributed to design of the experiment, manuscript preparation and determination of 8-oxodG in human blood. MH-R contributed to writing the manuscript, procurement of external funding and study design. DJ contributed to study design, manuscript preparation and data presentation. All authors read and approved the manuscript.
